# To-Day and To-Morrow

**Published:** 1906-06-30

**Authors:** 


### TO-DAY AND TO-MORROW.

The King of the Belgians has sent a donation of twenty guineas to the St. John's Hospital for Diseases of the Skin.

Guy's Hospital garden party will be held on July 4th, and Sir W. Cameron Gull will distribute the medals and prizes at 3.15 p.m.

The festival dinner of the Royal Hospital for Incurables, Putney, is fixed for July 6th at the Savoy Hotel; Mr. H. J. Allcroft, the treasurer, will preside.

Queen Alexandra has given £50 to the Chapel Endowment Fund of the British Home for Incurables, the garden party in support of which will be held at the Home on July 14th at 2.30. The Bishop of Kingston, Dr. Hook, will speak.

The Association of Headmistresses in conference demand a better intellectual and physical standard for scholars passing from elementary into secondary schools. They urge the Board of Education to insist on the medical inspection of all successful condidates for scholarships before awarding them. The inspection should precede, not follow, success in competition.

